# Three-Dimensional Printing Enabled Droplet Microfluidic Device for Real-Time Monitoring of Single-Cell Viability and Blebbing Activity

**DOI:** 10.3390/mi14081521

**Published:** 2023-07-28

**Authors:** Meiai Lin, Ting Liu, Yeqian Liu, Zequan Lin, Jiale Chen, Jing Song, Yiya Qiu, Benqing Zhou

**Affiliations:** 1 Department of Biomedical Engineering, College of Engineering, Shantou University, Shantou 515063, China; meiailin@stu.edu.cn (M.L.); 20yqliu1@stu.edu.cn (Y.L.); 20jlchen@stu.edu.cn (J.C.); 20jsong@stu.edu.cn (J.S.); 20yyqiu@stu.edu.cn (Y.Q.); 2 Department of Biology, College of Science, Shantou University, Shantou 515063, China; 21tliu1@stu.edu.cn

**Keywords:** 3D Printing, droplet microfluidics, cell viability, cell blebbing

## Abstract

Droplet-based microfluidics with the characteristics of high throughput, low sample consumption, increasing reaction speed, and homogeneous volume control have been demonstrated as a useful platform for biomedical research and applications. The traditional fabrication methods of droplet microfluidics largely rely on expensive instruments, sophisticated operations, and even the requirement of an ultraclean room. In this manuscript, we present a 3D printing-based droplet microfluidic system with a specifically designed microstructure for droplet generation aimed at developing a more accessible and cost-effective method. The performance of droplet generation and the encapsulation capacity of the setup were examined. The device was further applied to measure the variation in cell viability over time and monitor the cell’s blebbing activity to investigate its potential ability and feasibility for single-cell analysis. The result demonstrated that the produced droplets remained stable enough to enable the long-time detection of cell viability. Additionally, cell membrane protrusions featuring the life cycle of bleb initiation, expansion, and retraction can be well-observed. Three-dimensional printing-based droplet microfluidics benefit from the ease of manufacture, which is expected to simplify the fabrication of microfluidics and expand the application of the droplet approach in biomedical fields.

## 1. Introduction

Droplet microfluidics, appearing as one prominent branch of microfluidics, have been demonstrated as a promising technology to be extensively used in biomedical fields because of their significant advantages [1,2,3]. Droplet microfluidics can provide an ultrasmall and isolated chamber with the features of reducing the risk of cross-contamination, low sample consumption, and high-throughput detection, facilitating the development of single-cell analysis [4,5,6,7]. For example, droplet microfluidic devices have been developed for single-cell sequencing, such as DNA sequence analysis [8] and RNA sequencing [9,10]. Single-cell secretion analysis is another one of the compelling application fields of droplet microfluidics to obtain information about specific cellular functions caused by the cell heterogeneity of diseases [7,11]. For example, Abate et al. reported an integrated droplet microfluidic device with antibody barcodes allowing for multiplexed single-cell protein secretion profiling [12]. Furthermore, the rapid generation of droplets enables parallel experiments and high-throughput measurements with a reduction in costs and enhancement of analytical efficiency, which can be readily adapted for drug screening [13]. In addition, the droplet can function as an isolated microreactor with a precisely controlled volume, rapid transport, and the mixing of reagents, providing a readily effective tool to study biochemical reactions, biomaterial synthesis, and cell culture [5,14,15,16].

There are various manufacturing approaches available for the fabrication of droplet microfluidic devices, differing in the manufacturing process, material, cost, and minimum feature sizes. Polydimethylsiloxane (PDMS)-based microfluidics utilizing soft lithography to fabricate microchannels are most commonly used due to the merits of gas permeability, relatively low costs, and flexible microstructure design at the nanoscale [17,18]. However, the problem is that they necessitate the steps of molding, aligning and sealing, which are time-consuming and dependent on specialized skills, leading to poor reproducibility. Moreover, it is considerably burdensome to achieve complicated 3D structures on a PDMS-based microfluidic chip [19]. Recently, ultrafast laser-based fabrication enables the fast prototyping and micromachining of devices with complex three-dimensional structures. The advantages lie in its high precision and the lack of restriction of substrate materials though the cost is relatively high [20,21]. Other alternatives include fabrication based on the inorganic materials of glass or silicon using wet-etching techniques and photolithography techniques, with the advantages of high optical transparency, mechanical rigidity, and good solvent compatibility [22]. However, the fabrication process necessarily requires micromachining, micromilling, hot embossing, and injection molding, due to the sophisticated instrumentation and tedious manual operation required [18,23]. 

In recent years, 3D printing technology has been increasingly developed and is becoming a promising alternative for improving the fabrication of microfluidics [24,25]. The superior advantage of 3D printing lies in the ability to convert a designed model, even if it has a 3D sophisticated architecture in the device, into a manufactured object in a single process under precise digital control. Compared to conventional fabrication methods, the designs for 3D printing-based microfluidics are more easily programed and reconfigured, permitting repeated performance testing and parameter optimization [26,27]. Additionally, the cost of 3D printing can be relatively low as the materials of only resin and solvent to remove support materials are needed and the fabrication procedure does not require an ultraclean environment, potentially making mass production and translation into commercial outcomes simpler. Three-dimensional printing-based droplet generators with various microstructures have been reported [28,29,30]. For instance, Ghaznavi et al. presented a monolithic 3D-printed axisymmetric co-flow structure to produce single and compound droplets [31]. Vijayan and Hashimoto combined 3D-printed fitting and commercially available needles to build a “plug-and-play” droplet generator [32]. Alessandri and colleagues designed a 3D-printed co-extrusion device to encapsulate human neural stem cells differentiating into neurons within capsules [33]. However, in comparison with other droplet generator methods, the application of a 3D printing-based droplet generator is restricted by the limited resolution of 3D-printed structures since currently most available printing methods can only produce channels larger than one hundred micrometers [18]. Additionally, the surface roughness as well as the optical transparency of printing material also become concerning issues. As an emerging technology, 3D printing-based droplet microfluidics still need to be further explored and developed to expand their application fields.

In this manuscript, we present a 3D-printed microfluidic device with specifically designed microchannels and examine the feasibility of the generated droplets for single-cell analysis. A droplet generator with a customized 3D microstructure enabling the flow-focusing of the continuous phase and dispersed phase for droplet generation was designed and printed by a 3D printer. We assembled the droplet generator with glass capillaries to enhance optical transparency to visualize droplet generation. The performance of the microfluidic system for the generation of droplets with a controllable size and product rate was validated. Additionally, the ability of the droplet device for encapsulating single particles was examined. We investigated the feasibility of the droplets for single-cell analysis by monitoring cell viability and tracking the cell’s blebbing activity. The 3D printing-based droplet microfluidics benefit from their ease of fabrication, which is expected to simplify the fabrication of microfluidics and thus expand the application of the droplet technique in biomedical fields such as 3D cell culture, biochemical microreactors, and drug carriers.

## 2. Materials and Methods

### 2.1. Design and Fabrication of the Droplet Microfluidic Device

The fundamental aspect of droplet production is to utilize the interfacial tension and shear forces at the interface between immiscible liquids, functioning as continuous-phase fluid and dispersed-phase fluid, respectively, to pinch off the stream to form droplets with controllable sizes. Microfluidic structure is one of the critical factors that determine the generation and properties of droplets such as size and product rate. In this work, a droplet generator was designed based on the co-flowing strategy and fabricated by using 3D printing technology. 

**Figure 1:**
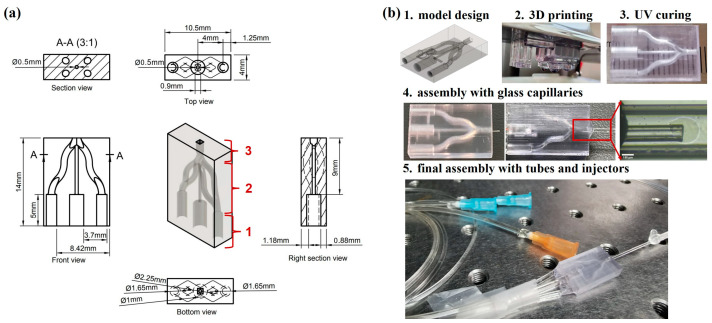
(**a**) Structure of the 3D-printed microfluidic device. The 3D model located in the center consists of (1) the connection section, (2) the separating flow section, and (3) the converging flow section. The section view, top view, side view, back view, and front view of the model are shown simultaneously. (**b**) Illustration of the fabrication procedure of the droplet microfluidic device, including model design, 3D printing, UV curing, and assembly with glass capillaries, tubes, and injectors. The microscopic image shows the co-axial of two glass capillaries.

The main component of the microfluidic device is a printed droplet generator with a specific 3D microstructure. Figure 1a detailly illustrates the microstructure of the droplet generator from different views with labeled parameters, with the 3D model displayed in the center. The generator consists of three parts: (1) the connection section, (2) the separating flow section, and (3) the converging flow section. The connection part is designed to connect the external pipes to introduce fluids, which contains 3 cylindrical channels. The diameter of the central channel for dispersed-phase fluid is 2.25 mm, and that of the other two channels on each side is 1.65 mm for continuous-phase fluid. Note that the bilateral channels for continuous-phase fluid quickly bifurcate in the axial direction (as shown from the side view), with each channel splitting into two winding subchannels. The subchannels are introduced here aiming to generate relatively large and balanceable shear pressure around the dispersed phase in the 3D space at the co-flowing region. These subchannels can converge with the central channel in the converging flow section, with the formation of co-flowing phenomena by dispersed-phase and continuous-phase fluids. The outlet located at the end side of the generator allows the extra connection with other components such as the glass capillaries and tubes according to the practical application. 

Figure 1b shows a sketch of the fabrication procedure of the droplet microfluidic device, which includes model design, 3D printing, UV curing, and microfluidics assembly with glass capillaries, tubes, and injectors. Specifically, once the model design of the microfluidic chip is completed, the model document can be uploaded to a stereolithography 3D printer (Asiga, Alexandria, Australia) with a resolution of 10 μm for the next round of printing using clear resin as the printing material. Subsequently, the printed chip is cleaned by using isopropanol and an ultrasonic washer to remove the residual resin, and then exposed to ultra-violet radiation for curing. Finally, the printed chip is assembled with glass capillaries, tubes, and injectors to obtain a complete microfluidic device allowing droplet production. To enhance optical transparency to clearly visualize the co-focusing effect, two glass capillaries with different widths can be optionally inserted into the central chip through the outlets in this work. As the magnified image shows in Figure 1b, the two glass capillaries can keep the co-axial allowing the inducement of co-flowing of the continuous-phase and dispersed-phase fluids. 

### 2.2. Experimental Setup

**Figure 2:**
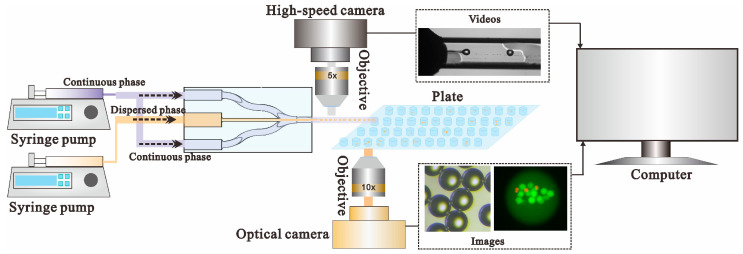
Schematic view of experimental setup. Two syringe pumps are connected to the printed generator device to drive the fluids into the microfluidics. A cell plate is used to collect the droplets. Additionally, a high-speed camera is used to record the dynamic process of droplet generation via a 5× objective lens. Additionally, a CMOS camera is employed to image the collected droplets and cells. The experimental data can be uploaded to a computer for further analysis.

The schematics of the experimental setup are shown in Figure 2. Two syringe pumps connected with the microfluidic device imposed constant volumetric flow rates to drive the continuous-phase fluid and dispersed-phase fluid, respectively. The droplets generated from the microfluidic device were collected using a common 96-cell plate. During the experiments, to keep the droplet stable, an appropriate amount of continuous-phase liquid was added to each pore of the 96-cell plate for droplet collection. A high-speed camera (Mikrotron, Unterschleißheim, Germany) with a frequency of 1 kHz was employed to record the dynamic process of droplet generation via a 5× objective lens. Additionally, a color CMOS camera (Thorlabs, Newton, NJ, USA) was equipped on an inverted optical microscope (Olympus, Tokyo, Japan) to image the collected droplets and cells. The captured videos and images were then uploaded to a computer for further analysis. Note that, in this study, we mainly focus on the applications of a droplet microfluidic system for the generation of water-in-oil droplets; thus, mineral oil with an added surfactant was chosen as the continuous-phase liquid, and an aqueous solution such as phosphate-buffered saline (PBS) solution or a cell suspension was used as the dispersed-phase liquid. 

### 2.3. Sample Preparation

Mineral oil (Sigma-Aldrich, St. Louis, MO, USA) was used as the continuous-phase fluid and the surfactant of span80 with 1% concertation was added to stabilize the droplets through the spontaneous accumulation at the droplet−carrier phase interface. The dispersed-phase fluids used in this work included PBS solution, microsphere solution, and cell suspension. The PBS solution was used as dispersed-phase fluid in the experiment of the performance test for droplet generation. A solution of 9.9 μm fluorescent microspheres, which could be excited at a wavelength of 540 nm and emitted at 560 nm wavelength, with a concentration of about 1 × 10^6^/mL was used to examine the capability of droplet encapsulation, and the triple-negative breast cancer cell line MDA-MB-231 was cultured and used in the experiments to measure cell viability and monitor cell’s blebbing activity. The cell suspension was prepared with a concentration of 1 × 10^6^/mL. The double staining of cells was performed by using the fluorescence dyes Calcein-AM and propidium iodide (PI) following the manufacturer’s instructions. PI ($\lambda_{ex}=530nm$, $\lambda_{em}=620nm$) is a nuclear staining reagent that can stain DNA and is often used to detect apoptosis since it can cross the damaged rather than the viable cell membrane. The dye Calcein-AM ($\lambda_{ex}=490nm,\lambda_{em}=515nm$) is a cell-staining reagent that can easily penetrate the membrane of living cells and thus fluorescently label living cells. The combination use of Calcein-AM with PI enables the fluorescent identification of live and dead cells simultaneously. The cultured cells of MDA-MB-231 were digested by trypsin and centrifuged with 1200 rpm/min to collect the cell suspension. Then, the dyeing working solution containing 20 μL of Calcein-AM, 40 μL of PI and 2.5 mL of PBS was added to the cell suspension. After 15 min of incubation, the cell suspension with double staining could be well-used for experiments. 

## 3. Results 

### 3.1. Droplet Generation

**Figure 3:**
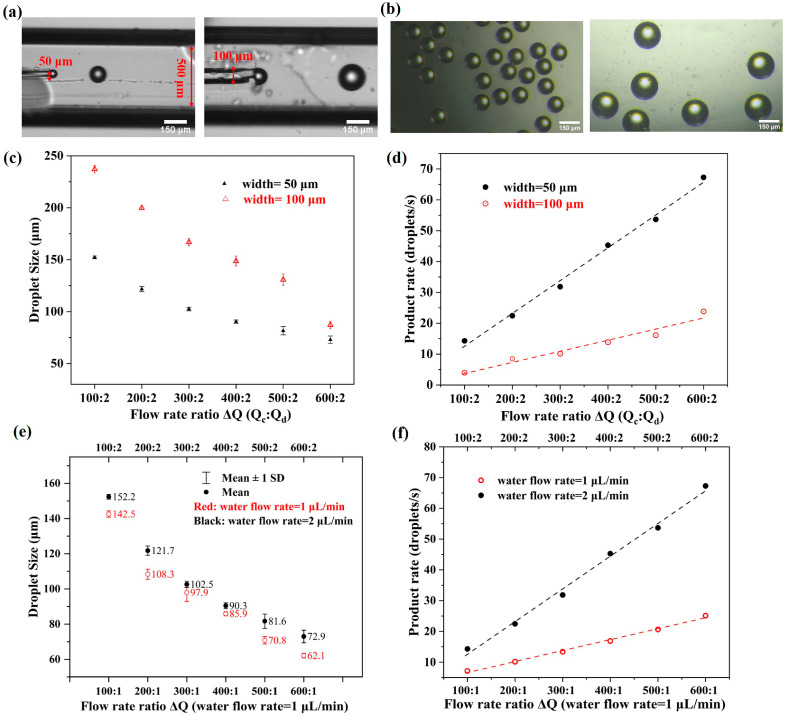
(**a**) Microfluidic channels with the circular glass capillaries of 50 μm and 100 μm in diameter. (**b**) Microscopic images of the droplets generated by the corresponding microfluidic devices in (**a**). (**c**) Droplet size varying with the flow rate ratio of the oil phase ($Q_{c}$) and the PBS phase ($Q_{d}$) from the different microfluidic devices. (**d**) Product rate of droplets varying with the flow rate ratio, ∆Q, of $Q_{c}$ and $Q_{d}$. (**e**) Droplet size varying with ∆Q when $Q_{d}$ is set as 1 μL/min and 2 μL/min using the device with glass capillaries of 50 μm. (**f**) Product rate varying with ∆Q when $Q_{d}$ is set as 1 μL/min and 2 μL/min using the device with glass capillaries of 50 μm.

Since droplet formation is mainly determined by the microchannel structure and fluid properties such as composition, viscosity, and flow rates, we investigated how these factors influence the droplet size and product rate, respectively. First, to investigate the effect of microchannel width on droplet generation, the circular glass capillaries with an inner diameter of 50 μm and 100 μm were inserted into the central channel of each printed generator for dispersed-phase fluid, respectively. Meanwhile, a square glass capillary with 500 μm in diameter was also used to connect with the converging-flow section of the printed chip to keep coaxial with the central glass capillary, allowing for the co-flowing of the fluids. For this experiment, the mineral oil with 1% span80 surfactant and PBS solution were used as continuous-phase fluid and dispersed-phase fluid, respectively. By controlling the flow rates of syringe pumps, the liquid interface of the continuous phase and dispersed phase can be disturbed as well as the shear pressure, causing droplet breakup. Figure 3a displays the moment of droplet formation for each droplet microfluidic device, with one pinched droplet flowing ahead and the next one forming at the outlet of the inner channel. The corresponding generated droplets were collected separately with a 96-well cell plate. As shown in Figure 3b, the droplets in each image present a distinct outline and uniform size. The droplets in the left image for the device with a 50 μm channel are smaller than those in right one for the device with a 100 μm channel. 

Then, the quantitative analysis of droplet size and product rate measured with controlling flow rate ratios, ∆Q, for each case was performed. From the result of droplet size distribution in Figure 3c, we can see that the droplet size decreases with the increasing flow rate ratio, ∆Q, of the continuous phase, $Q_{c}$, and dispersed phase, $Q_{d}$, which is fixed as 2 μL/min for both cases, while for the device with a 100 μm channel, the droplets are about 1.5 times larger than those produced by the device with a 50 μm channel under the same flow rate ratio. The comparison result of the product rate is shown in Figure 3d, which demonstrates that an increasing flow rate ratio can lead to a rising product rate. In addition, the device with a thinner channel can generate droplets more quickly. This is reasonable and within expectation since a larger difference in the cross-section of the co-flowing channel and dispersed channel can cause higher shear stresses to pinch off the fluid faster and thus produce droplets more easily.

Next, the flow rate of the dispersed phase, $Q_{d}$, is set as 1 μL/min and 2 μL/min, respectively by using the microfluidic device with glass capillaries of 50 μm to examine its influence on droplet generation. It can be found from Figure 3e,f that both the droplet size and product rate tend to generally increase with an increasing dispersed phase flow rate. This makes sense because it is possible for a greater volume of the dispersed phase to enter the droplet before it is snipped from the generation region.

### 3.2. Encapsulation of Single Particles

**Figure 4:**
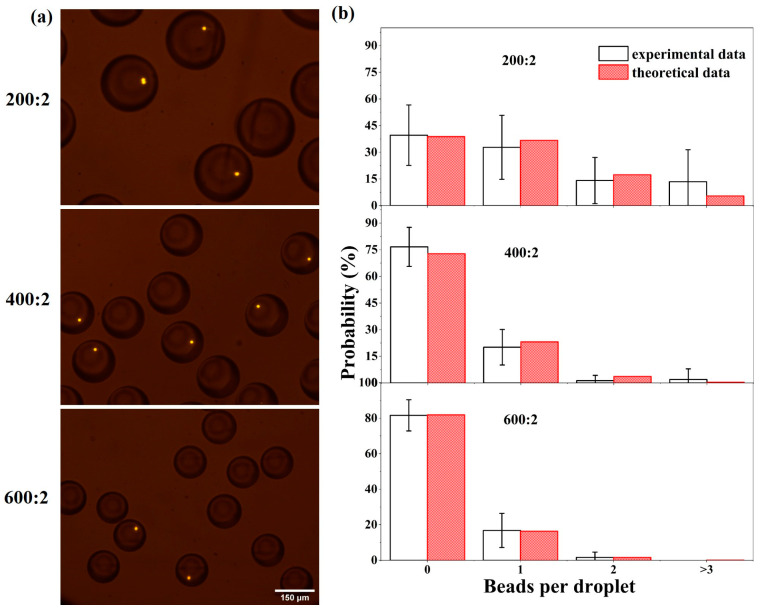
(**a**) The images of droplets encapsulating the fluorescent beads under various flow rate ratios. (**Top**): 200:2; (**Middle**): 400:2; (**Bottom**): 600:2. (**b**) Probability of different numbers of beads within each droplet, corresponding to the flow rate ratios of 200:2, 400:2, and 600:2.

The encapsulation capacity is essential for droplets to provide a separated microchamber with a controllable volume for application in single-cell analysis, material synthesis, and biochemical reactions. Hence, to test the potential ability of our droplet microfluidic system for encapsulating single particles and reagents, the solution of fluorescent beads (Thermo Fisher, Waltham, MA, USA) was used as the dispersed-phase fluid driven by the syringe pump at a flow rate of 2 μL/min. By changing the flow rate of the oil phase with 1% span80, the various droplets encapsulating different numbers of beads could be collected. Figure 4a shows the images of droplets within fluorescent beads generated at various flow rate ratios, ∆Q, of 200:2, 400:2 and 600:2 from top to bottom, respectively. As expected, the size of the droplets reduces gradually when the ∆Q becomes larger. 

According to the previous publication [15] the bead encapsulation in droplets follows the Poisson distribution, which is given by

(1) $$P\left(\lambda ,k\right)=\frac{\lambda^{k}e^{-\lambda }}{k!}$$

where λ is the average number of particles in each droplet, k is the number of particles in a droplet and P is the probability of k particles in a droplet. Since λ is proportional to the volume of the droplet and the concentration of the sample solution, the decrease in droplet size will lead to more droplets without any beads. Figure 4b shows the comparison of measured results and theoretical data for the different experimental conditions. The experimentally determined values maintain in good agreement with the theoretical estimates. To be specific, for the case with a ∆Q of 200:2, the droplets with a single particle account for over 30%, and the percentage of empty droplets is close to 40%. When the flow rate ratio increases, the encapsulation rate for single particle reduces by 10% for ∆Q=400:2 and by about 15% for ∆Q=600:2. In contrast, the increasing flow rate ratio can cause the generation of more empty droplets. The probability grows up from about 40% to approximately 80% for ∆Q=600:2. Furthermore, when the number of beads in a droplet increase, the corresponding probability will decline quickly. Based on the above result, ∆Q of 200:2 is preferable for encapsulation reagents or particles due to the relatively low probability of empty droplets. It should be noted that the theoretical value of λ is calculated by referring to the real average number of particles per droplet, which would be less due to particle sedimentation and aggregation. To summarize, the result suggests that it is achievable to apply droplet microfluidics for further application involving single-cell reagent encapsulation. 

### 3.3. Measurement of Cell Viability 

The cell viability within a droplet is important, especially for cell culture and drug screening, which requires the droplets to maintain steady enough for a long time. Here, we used the triple-negative breast cancer cell line MDA-MB-231 for the experiment to measure cell viability over time. The suspension of stained cells was introduced into the microfluidic system as the dispersed-phase fluid and the mineral oil with 1% span80 was used as the continuous-phase fluid. The flow rate ratio, ∆Q, was kept as 200:2. The collected droplets with stained cells were imaged by a CMOS camera (Thorlabs, Newton, NJ, USA) via a 10× objective lens.

**Figure 5:**
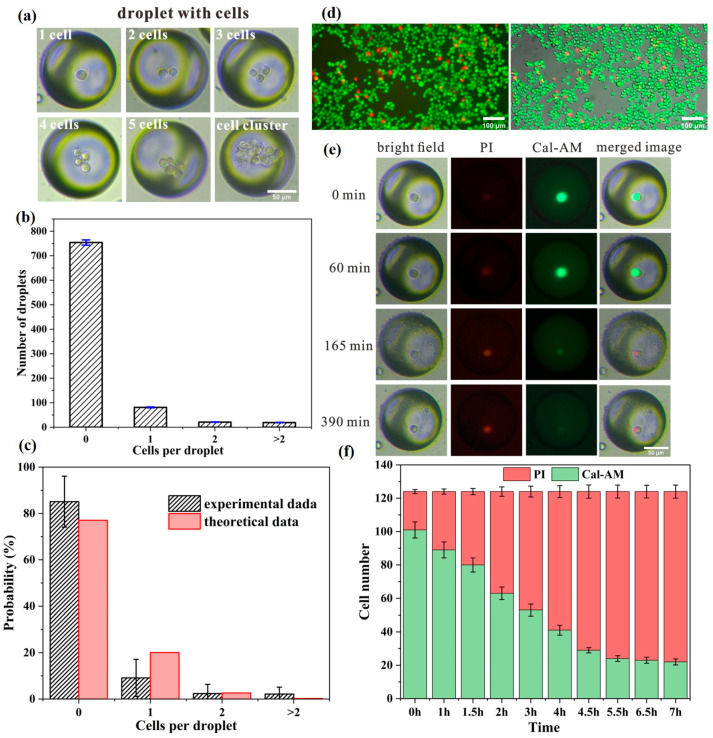
(**a**) Bright-field images of droplets encapsulating different numbers of cells. (**b**) Statistical result of different numbers of cells per droplet. (**c**) Probability distribution of cell encapsulation. The black histograms indicate the experimental data and red histograms refer to theoretical result. (**d**) Images of breast cancer cells stained by PI (red) and Calcein-AM (green). Left image: merged image of the red channel and the green channel. Right image: merged images of the red channel, the green channel, and the bright-field image. (**e**) Different modes of a droplet encapsulating a single cell over time. First column: bright-field images at time points of 0 min, 60 min, 165 min, 390 min. Second column: red channel of PI staining. Third column: green channel of Calcein-AM staining. Last column: merged images of the three channels. (**f**) Measurement of cell viability over time.

First, the cell encapsulation probability of droplets was measured and analyzed. Figure 5a presents the microscopic images of uniform droplets encapsulating different numbers of cells: one cell, two cells, three cells, four cells, five cells and a cell cluster that contains more than five cells. The statistical result of the cells encapsulated by each droplet is shown in Figure 5b. Additionally, the probability distribution of cell encapsulation is calculated and compared with the theoretical result in Figure 5c. We can see that the number of droplets with a single cell is smaller than the theoretical result. In contrast, the droplets with more than two cells appearing as cell clusters are beyond the expected value. This can be attributed to the fact that biological cells have considerably high surface adhesion and thus are more likely to aggregate together instead of distributing individually leading to cell sedimentation. 

Next, we applied the droplet microfluidic system to measure cell viability. Figure 5d presents the merged images of breast cancer cells simultaneously stained by PI in red and by Calcein-AM in green. The fluorescent image on the left is obtained by merging the images from the red channel and green channel. Additionally, the one on the right is the merged result of Calcein-AM-stained, PI-stained, and bright-field images. Obviously, almost all cells are successfully stained by the fluorescent dye. Furthermore, at the initial preparation, most of the cells are active and shown in green with only a fraction of cells (<5%) presented in red which can be regarded as dead. To visualize the changes in cell viability, the images of the same droplets were captured at different time points with a time interval of about 30 min. Note that about 30 min is required to prepare and complete the collection of droplets before imaging. Thus, to avoid ambiguity in time labeling, we specify the time point when the first image of a droplet is obtained as the initial time. The duration of the experiments was about 9 h to measure cell viability.

Figure 5e displays the different imaging modes of a droplet encapsulating only one cell over time. The images in the first column are obtained under the bright-field mode at different time points. The images in the second column indicate the intensity of PI and the intensity of Calcein-AM is shown in the third column. The different modes of images are merged to reveal the real-time changes in cell viability within the droplet. From the result, we can see that the living cell has quite a strong fluorescence intensity of Calcein-AM and a dark PI intensity at the initial time point. Then, the fluorescence intensity of PI gradually increases while that of Cal-AM slowly reduces over time. This means that the cell is gradually losing its viability. After 390 min, it seems that the cell becomes almost dead since the signal of Calcein-AM appears too weak to be detected. This demonstrates that the cells can survive within the droplets for a period but then become inactive and finally lose activity. Figure 5f shows the long-time measurement of cell viability changing over time within 7 h. The number of living cells almost linearly reduces from about 81% to 23% after about 4.5 h. Additionally, the fraction of inactive cells rises to more than 80% after 7 h.

### 3.4. Real-Time Monitoring of Cell Blebbing

Cell blebbing is a dynamic process involving spherical membrane protrusions, which has been reported that usually can be observed during apoptosis, cytokinesis, and even migration in three-dimensional cultures. Especially, for tumor cells, the blebbing migration seems to be a common alternative to the more extensively studied lamellipodium-based motility [34,35]. To investigate the stability of droplets for real-time tracking and visualizing the dynamic process of cell blebbing, the breast cancer cells simultaneously stained by Calcein-AM and PI within the droplets were used to perform experiments with the steps mentioned previously. 

**Figure 6:**
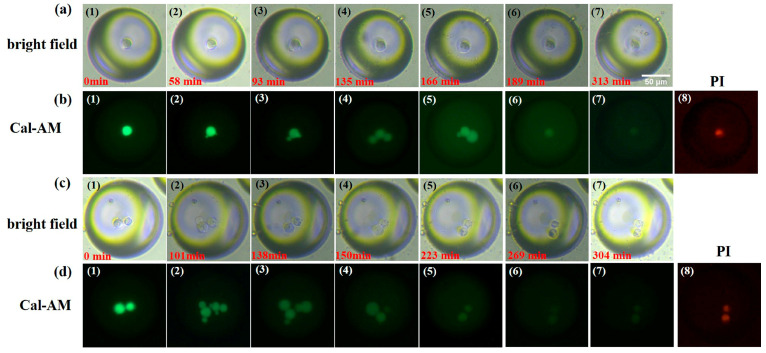
(**a**) The bright-field images of one cell over time: (**1**) 0 min, (**2**) 58 min, (**3**) 93 min, (**4**) 135 min, (**5**) 166 min, (**6**) 189 min, and (**7**) 313 min. ((**b**) (**1**–**7**)) The fluorescent images of Calcein-AM staining of a cell imaged over time corresponding to ((**a**) (**1**–**7**)). ((**b**) (**8**)) The PI channel of the cell at 313 min of ((**a**) (**7**)). (**c**) The bright-field images of two cells over time: (**1**) 0min, (**2**) 101 min, (**3**) 138 min, (**4**) 150 min, (**5**) 223 min, (**6**) 269 min, and (**7**) 304 min. ((**d**) (**1**–**7**)) The fluorescent images of Calcein-AM staining of a cell imaged over time corresponding to ((**b**) (**1**–**7**)). ((**d**) (**8**)) The PI channel of the cell at 313 min of ((**c**) (**7**)).

Figure 6 shows the typical cell images obtained at different modes. The bright field images of droplets with single and two cells are presented in Figure 6a,c, respectively. Additionally, the images of (1)–(7) in both Figure 6a,c correspond to different time points as labelling at the bottom of each image. Although the morphology of cells such as the membrane protrusions and blebbing cannot be clearly imaged by using bright field mode due to the limited resolution and low contrast, the general changes in relative spatial positions of the two cells can be observed in Figure 6c. The corresponding fluorescence images of Calcein-AM and PI are presented in Figure 6b,d. Interestingly, the membrane protrusion of blebbing activity can be well imaged and tracked in the green channel. From Figure 6b, the single cell appears considerable circular shape without any obvious blebbing at the initial. Then, there are two small protrusions emerging on the cell surface, which is described as bleb initiation. These blebs continue to grow up and expand with the diameter reaching about 13 μm at 135 min as shown in Figure 6b(4). This dynamic process is named bleb expansion. What is even more interesting is that at 166 min, one of the blebs enters the retraction process, with the diameter decreasing to 8 μm, whereas another one continues to expand to about 15 μm. However, at the time point of 189 min (Figure 6b(6)), there is only one cell that can be detected, without any other bleb or vesicle. Then, the intensity of Cal-AM becomes too weak to track, which can be attributed to the fluorescent bleaching and the loss of cell viability, while the image of PI staining corresponding to the time point of 313 min represents an increasing intensity, as shown in Figure 6b(8). This suggests the cell is almost inactive and even dead. 

For the case of the droplet encapsulating two cells, a similar life cycle of bleb initiation, expansion, and retraction can also be observed. As shown in Figure 6d, there are several protrusions on each cell’s membrane. Additionally, some blebs expand with other blebs retracting. A significant variation in bleb number can be observed are appear throughout the whole process. At the time point of 101 min, there are five blebs appearing. Then, the number of blebs reduces to three at 138 min (Figure 6d(3)), and there is only one left at 150 min (Figure 6d(4)). At the time point of 223 min (Figure 6d(5)), the blebs disappear with the original two cells left, due to bleb retraction and fluorescent bleaching. After that time point, the intensity of Calcein-AM becomes quite faint, whereas the corresponding PI intensity enhances gradually as shown in Figure 6d(8), which indicates a decline in the cell viability. 

## 4. Discussion 

In this work, we designed a 3D-printed droplet generator with winding subchannels for the continuous phase instead of the straight structures commonly used, causing a higher shear force to be exerted by the continuous phase on the dispersed phase to produce droplets faster under the same flow rate ratio. Additionally, the 3D-printed material used here is a kind of clear resin, enabling the inner microstructure to be visualized to some extent. However, it is still not transparent enough to allow us to image the generation process of droplets within the chip clearly. In addition, although the resolution of a 3D printer is 10 μm, which in theory permits the fabrication of microchannels at the micron level, the printing of microchannels with a width below 100 μm frequently suffers from the problem of blockage in practice. To address these issues, we adopted the approach of assembly with glass capillaries to provide enough transparency for a visualization of the dynamic process of droplet generation. Additionally, the glass capillary with a width of several microns is commercially available, compensating for the 3D printing. As mentioned above, the glass capillaries of 50 μm, 100 μm, and 500 μm as alternative microchannels were integrated with the printed chip. The limitation of this capillary-based approach lies in the possibility of artificial assembly to introduce an inaccuracy in alignment, which may cause deviation from the central line. Moreover, the sealing glue to immobilize capillaries may fall off during the experiment due to the fluid’s impact. If the issues of transparency and channel size are not concerned in the practical applications, then the use of 3D printing-based microfluidics without glass capillaries is enough to meet the requirement, which can further simplify the fabrication process of microfluidics. 

We applied droplet microfluidics to encapsulate microspheres and biological cells. In terms of the probability of encapsulation which follows the Poisson distribution, the critical parameter λ, the average number of particles per droplet, in theory, depends on the droplet volume and concentration of dispersed-phase fluid. However, the actual concentration of fluid is usually lower than the expected value due to sample sedimentation during the experiment. Therefore, the measured value of λ may be smaller than that in the calculated data. In this manuscript, the total number of encapsulated microspheres is counted and divided by the total amount of droplets to obtain the theoretical value of λ. As aforementioned, the decrease in droplet volume will give rise to more empty droplets as fewer samples can be driven into droplets. Thus, it is important to control the parameters of the droplet microfluidic system to generate droplets with desirable sizes. Furthermore, the droplet size and volume decide the amount of culture media containing the nutrients, amino acids, and growth factors, which are associated with the ability of the cell to survive [36]. The volume of droplets encapsulating cells is about 1 nL in this work. For one cell in a droplet, this concentration is equivalent to 10^6^ cells/mL which is considerably high. Undoubtedly, the living space becomes very crowded for cell clusters, which could speed up the rate of reduction in cell viability, as discussed in the reference [15]. It should be noted that in this work we only measured cell viability varying with time to test the potential ability of droplets to support cell survival, whereas we did not distinguish the differences in the cell inactivation rate for droplets encapsulating various cells. Therefore, the droplet size should be carefully selected and controlled in terms of specific applications.

We also presented the blebbing activity with the time evolution of single cells within droplets, which features the dynamic life cycle of bleb initiation, expansion, and retraction. Compared to other microfluidics-based cell tracking methods, such as the “confined microchannel” strategy [37], “parallel chamber arrays”-based microfluidics [38] and the “well arrays”-based cell capture approach [39], the 3D printing-based droplet microfluidic approach we adopted here has the advantages of high throughput and flexible volume control. Cell blebs have been reported to be observed during cell apoptosis, cytokinesis and even cell spreading on both 2D and 3D substrates [35], though the specific mechanism of action remains to be clarified. We reckon that the behavior of cell blebs appearing in our observation can be attributed to the execution phase of apoptosis as the cells gradually become inactive and finally die over time. This may be attributed to the fact that the droplet-encapsulated MDA-MB-231 cells in suspension may die due to the anoikis phenomenon when the cells are detached from the extracellular matrix [40]. In addition, the fluorescence dye of Calcein-AM has been reported to be toxic to several cell lines, which may also induce cell death [41]. For future work, more experiments need to be performed to further explore the exact cause of bleb activity.

## 5. Conclusions

In this manuscript, we proposed a 3D printing-based droplet microfluidic device with a specifically designed microstructure for droplet generation. The effects of microchannel structure and flow rates on droplet size and product rate were discussed in detail. Then, the device was further applied for cell encapsulation, the long-time measurement of cell viability, and real-time monitoring of the cell’s blebbing activity. Our device benefits from its simplicity and is expected to offer an alternative to simplify the fabrication of conventional microfluidics to expand the application of the droplet approach in biomedical fields such as cell culture, biochemical reaction, and drug screening.

## Data Availability

Data will be made available on request.
